# Light Color‐Controlled pH‐Adjustment of Aqueous Solutions Using Engineered Proteoliposomes

**DOI:** 10.1002/advs.202307524

**Published:** 2024-02-11

**Authors:** Daniel Harder, Noah Ritzmann, Zöhre Ucurum, Daniel J. Müller, Dimitrios Fotiadis

**Affiliations:** ^1^ Institute of Biochemistry and Molecular Medicine University of Bern Bern 3012 Switzerland; ^2^ National Centre of Competence in Research (NCCR) Molecular Systems Engineering Basel Switzerland; ^3^ Department of Biosystems Science and Engineering ETH Zürich Basel 4056 Switzerland

**Keywords:** bacteriorhodopsin, light‐driven proton pump, proteoliposome, proteorhodopsin, synthetic biology

## Abstract

Controlling the pH at the microliter scale can be useful for applications in research, medicine, and industry, and therefore represents a valuable application for synthetic biology and microfluidics. The presented vesicular system translates light of different colors into specific pH changes in the surrounding solution. It works with the two light‐driven proton pumps bacteriorhodopsin and blue light‐absorbing proteorhodopsin Med12, that are oriented in opposite directions in the lipid membrane. A computer‐controlled measuring device implements a feedback loop for automatic adjustment and maintenance of a selected pH value. A pH range spanning more than two units can be established, providing fine temporal and pH resolution. As an application example, a pH‐sensitive enzyme reaction is presented where the light color controls the reaction progress. In summary, light color‐controlled pH‐adjustment using engineered proteoliposomes opens new possibilities to control processes at the microliter scale in different contexts, such as in synthetic biology applications.

## Graphical abstract

This proteoliposome system translates light of different colors into specific pH changes of the surrounding solution. It works with the two light‐driven proton pumps bacteriorhodopsin (BR) and blue light‐absorbing proteorhodopsin Med12 (BPR), that are oriented in opposite directions in the vesicular lipid membrane. As application example, a pH‐guided enzyme reaction is shown where the light condition controls the reaction progress.

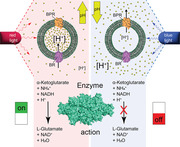

## 1 Introduction

Bottom‐up synthetic biology combines building blocks from nature and engineered versions thereof to new functional molecular systems and aims at their application in basic research, biotechnology, and medicine.^[^ ^1^ ^]^ Microbial rhodopsin proton pumps are membrane proteins that use light to translocate protons generally from the intracellular to the extracellular side of the plasma membrane, thereby powering the proton motive force of the cell.^[^ ^2^ ^]^ Thus, such light‐driven proton pumps represent excellent building blocks to energize bottom‐up assembled molecular systems. Microbial rhodopsin proton pumps consist of seven transmembrane α‐helices with amino‐ and carboxyl‐termini exposed to the extracellular and cytoplasmic sides, and a photoreactive retinal chromophore covalently linked through a Schiff base to a lysine residue in the seventh transmembrane α‐helix.^[^ ^3^ ^]^ Bacteriorhodopsin (BR)^[^ ^4^ ^]^ is found in the purple membrane (PM)^[^ ^4c^ ^]^ of the halophilic archaeon *Halobacterium salinarum (H. salinarum)*. BR absorbs primarily green light and was the first discovered light‐driven proton pump.^[^ ^4c^ ^]^ Its function and structure were studied extensively, and BR is widely applied in science and biotechnological applications due to its extraordinary chemical and thermal stability.^[^ ^5^ ^]^ Numerous homologous genes were found later in genetic isolates from various environmental sources throughout the microbial diversity, e.g., proteorhodopsins.^[^ ^6^ ^]^ The blue light‐absorbing proteorhodopsin Med12 (BPR)^[^ ^7^ ^]^ is one of them and can be produced efficiently in the bacterium *Escherichia coli (E. coli)*.^[^ ^7b^ ^]^

There are examples of the successful application of microbial rhodopsin proton pumps such as BR and proteorhodopsin for the light‐driven control of pH in aqueous solutions using pro‐ and eukaryotic cells,^[^ ^8^ ^]^ proteoliposomes,^[^ ^9^ ^]^ polymerosomes,^[^ ^10^ ^]^ and functionalized surfaces.^[^ ^11^ ^]^ From a bioenergetical and engineering point of view the combination of light‐driven proton pumps with ATP synthesis utilizing an established proton gradient across a membrane as a power source is an important achievement.^[^ ^12^ ^]^ To the best of our knowledge, the utilization of two proton pumps positioned in opposite orientations within the membrane for controlling proton gradients in synthetic systems has not been reported. This discovery has the potential to usher in a new chapter in understanding control options and the versatile applications of light‐dependent pH manipulation.

Here, we assembled bottom‐up functional BR and BPR into proteoliposomes that were utilized within a computer‐controlled feedback loop device that enables automated pH‐regulation of aqueous solutions by light of different wavelengths. Finally, the established system was consolidated and applied to control a pH‐dependent enzymatic reaction.

## 2 Results and Discussion

**Figure 1:**
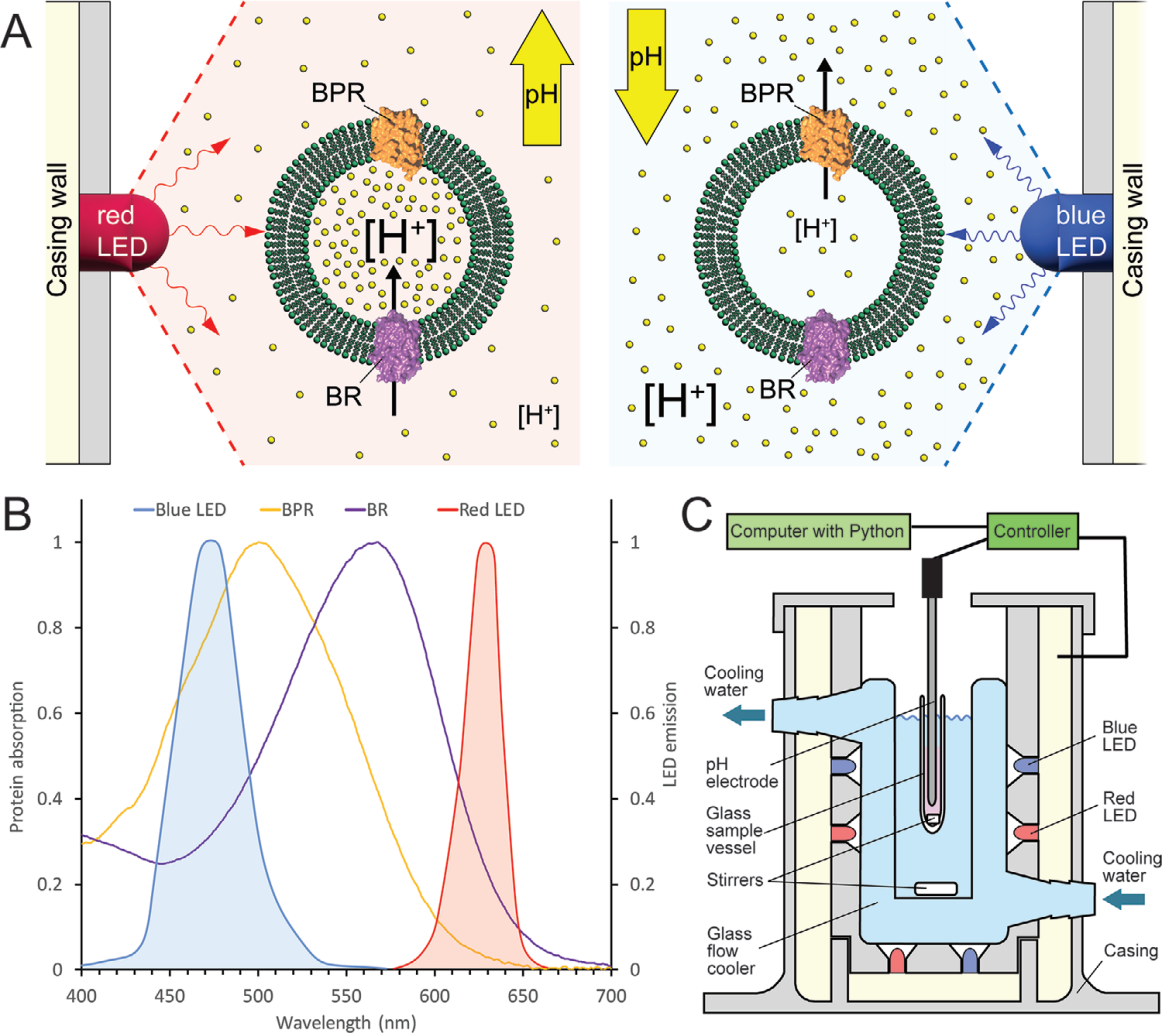
Measurement setup and spectral characteristics of bacteriorhodopsin (BR) and blue light‐absorbing proteorhodopsin Med12 (BPR). A) Schematic representation of proteoliposomes with the two proton pumps BR and BPR differently oriented in the vesicular lipid membrane. The two microbial rhodopsin proton pumps change the outside pH (yellow dots indicate protons) dependent on the applied light color due to the significantly different light absorption characteristics of BR and BPR. B) Absorption spectra of BR and BPR compared to emission characteristics of the used red and blue LEDs. C) Setup for pH determination and adaptation by changing light color conditions using a micro pH‐electrode. The illumination chamber with computer‐controlled red and blue LEDs contains the stirred measuring solution within a cooling water bath.

BR and BPR were expressed homologously or heterologously in *H. salinarum* or *E. coli*, respectively. Both light‐driven proton pumps were solubilized from corresponding cell membrane preparations using the detergent *n*‐octyl‐β‐D‐glucoside (OG), isolated and reconstituted into 1,2‐dioleoyl‐*sn*‐glycero‐3‐phosphocholine (DOPC) liposomes. Sodium dodecyl sulfate‐polyacrylamide gel electrophoresis (SDS‐PAGE) analysis indicated high purity of the two light‐driven proton pumps with bands migrating ≈20 kDa (Figure S1, Supporting Information). Reconstitution of BR or BPR into lipid bilayers yielded functional proteoliposomes pumping protons in opposite directions (Figure S2, Supporting Information). This observation indicated the predominant integration of the two light‐driven proton pumps in opposite directions into liposome membranes when using our reconstitution protocol. Based on this result, we co‐reconstituted BR and BPR together into DOPC membranes to obtain bifunctional proteoliposomes, i.e., vesicles being able to pump in both directions depending on the applied light color (**Figure** **1**A). Proteoliposomes containing both, BR and BPR had an average diameter of ≈130 nm and a concentration of ≈20 nM particles in the photoactivity assay, as determined by nanoparticle tracking analysis (Figure S3, Supporting Information). The orientation of BR and BPR in proteoliposomes (i.e., in proteoliposomes containing BR or BPR, or both) was quantified by carboxypeptidase digestion (Experimental Section). According to the observed pumping action (Figure S2, Supporting Information), the carboxyl‐termini of BR and BPR reconstituted into proteoliposomes should be exposed to the outside (extravesicular solution) and inside (proteoliposome lumen), respectively. SDS‐PAGE analysis of carboxypeptidase‐treated vesicles (Figure S1, Supporting Information) indicates that about two‐thirds of the BR carboxyl‐termini were digested (i.e., accessible to the carboxypeptidase, exposed to the outside), while no digestion of BPR carboxyl‐termini was observed (i.e., not accessible to the carboxypeptidase, exposed to the inside). Thus, this biochemical result supports the predominant and distinct orientation of BR and BPR within the vesicular lipid bilayer (Figure 1A), and the related, observed directional, light color‐dependent proton pumping (Figure S2, Supporting Information).

The chromatic absorptions of the two light‐driven proton pumps are significantly different. BPR absorbs light in the green range, shifting toward blue, while BR absorbs light in the green range, shifting toward red with absorption maxima of ≈498 and ≈568 nm, respectively (Figure 1B). Specific activation of one of the pumps can thus be achieved by using light colors in a spectral absorption range covered mainly by one of the light‐driven proton pumps while touching the absorption of the other only minorly. The predominant spectral emissions of the LEDs utilized in this study are illustrated in Figure 1B, and they primarily overlap with the absorption spectrum of either BR or BPR. Blue light activates mainly BPR, leading to an acidification of the solution outside the vesicles (Figure 1A, right). Red light activates mainly BR, raising the pH of the extravesicular solution (Figure 1A, left). Nevertheless, the blue light emission spectrum overlaps to some extent with the BR absorption spectrum (Figure 1B). Thus, blue light also activates BR, as observed in BR‐proteoliposomes, whereas red light does not activate BPR in BPR‐proteoliposomes (data not shown). The latter is in line with the observed minor overlap of the red light emission spectrum and the BPR absorption spectrum (Figure 1B). Consequently, the net pH effect observed with blue light results from the combined pumping activities of both light‐driven proton pumps. However, it is noteworthy that the pH change toward acidic is significantly dominated by BPR, as a 2:1 (weight to weight) ratio of BPR to BR was applied during reconstitution and the absorption maximum of BPR is optimally tuned to the blue light used in the experiment. To precisely control the pH in the proteoliposome solution, a device was designed consisting of a microcontroller connected to arrays of red and blue LEDs (Figure S4, Supporting Information), a micro pH‐electrode, and a computer connecting pH‐electrode and LEDs (Figure 1C). Temperature stability of the proteoliposome solution was maintained through water cooling and constant stirring in the sample vessel holding a volume of ≈150 µL. A feedback loop was established to automatically adjust the light color and intensity based on the difference between the measured pH and the selected pH. This device then controlled the light‐dependent proton pumping activities of BR or BPR to adjust the pH of the proteoliposome solution to the pH setpoint. The user can program the device to follow a series of predefined pH setpoints or change the pH setpoint in real‐time to the desired pH value. Moreover, the proportional and integral gains can be adjusted in real‐time to optimize the response characteristics.

**Figure 2:**
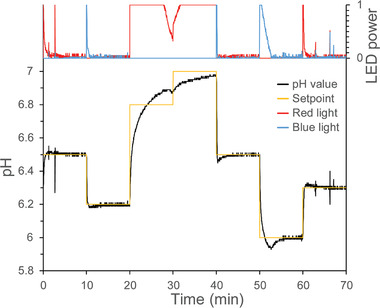
pH control and regulation of an aqueous solution by blue and red light using proteoliposomes with wavelength‐specific light‐driven proton pumps integrated in opposite directions in the lipid bilayer. Different setpoints are chosen (for 10 min, yellow line) and the software‐controlled feedback loop tunes the blue and red LED lights at variable intensities to reach and hold the selected pH (power level from 0 to 1 shown on the upper panel for the red and blue LEDs).

A representative experiment shows the described setup at work, controlling several setpoints between pH 6 and 7, with each setpoint being maintained for 10 min (**Figure** **2**). The upper panel of Figure 2 displays the LED activity controlled by the feedback loop. During phases of stable pH close to the setpoint, the LEDs are activated minorly. To change the pH toward a new setpoint, LED activity is maximal and drops quickly upon reaching the setpoint (Figure 2, 10 min). If reaching the setpoint takes longer, because of relatively large pH differences, full LED power can persist after reaching the setpoint due to the settings of proportional and integral gain in the feedback loop, thus needing gain optimization in that specific range (Figure 2, 20–30 min). If the pH is already close to the new setpoint upon setpoint change, LED power might be not maximal. LED power will increase due to the integral gain when the pH is not approaching the setpoint fast enough (Figure 2, 30–40 min). This happens particularly when the pH approaches the limits of the proteoliposomes, where they are not able to further pump protons against the established proton gradient: ≈pH 7 in Figure 2, where more time would be necessary to reach the setpoint. However, within most of the tested pH ranges the feedback loop was tuned optimally to reach the setpoint fast and hold it (using a proportional gain of 5 and integral gain of 0.1).

Next, the pH change limits and ranges of the system were tested. To this aim, proteoliposomes were washed in different pH solutions before measurement. The largest maximal ranges of more than two pH units were observed when starting at around neutral pH (Figure S5, Supporting Information right: Maximum pH range). The working pH range then decreases toward acidic and basic starting pHs, with evanescent activity at ≈pH 4 or 11. The temporal resolution of the pH change is summarized in Table S1 (Supporting Information), indicating generally faster pH changes through the activation of BPR. This faster pH change might be due to the ratio of BPR:BR of 2:1 (weight to weight) in the proteoliposomes, a generally higher performance of BPR, or the constellation of protein absorption spectra and LED emission peaks: The emission peak of the blue LED is broader than that of the red LED, resulting in a significantly larger overlap between the blue light and the BPR absorption spectrum (Figure 1B). Additionally, the emission maximum of the blue LED is closer to the absorption maximum of BPR when compared to the situation with red light for BR. The temporal resolution of the pH change shows also a prominently decreasing velocity when approaching the reachable pH limits of the setting. This means that fast changes take place in the first half of the maximally reachable pH step.

The glutamate dehydrogenase (GDH) enzyme reaction was then tested as a potential application of such a light‐controlled pH solution. The GDH catalyzes the reaction:

(1) $$\begin{array}{ccc} & & \alpha -\mathrm{Ketoglutarate}+{\mathrm{NH}}_{4}^{+}+\mathrm{NADH}+H^{+}\\ & & \;↔L-\mathrm{Glutamate}+{\mathrm{NAD}}^{+}+H_{2}O \end{array}$$

**Figure 3:**
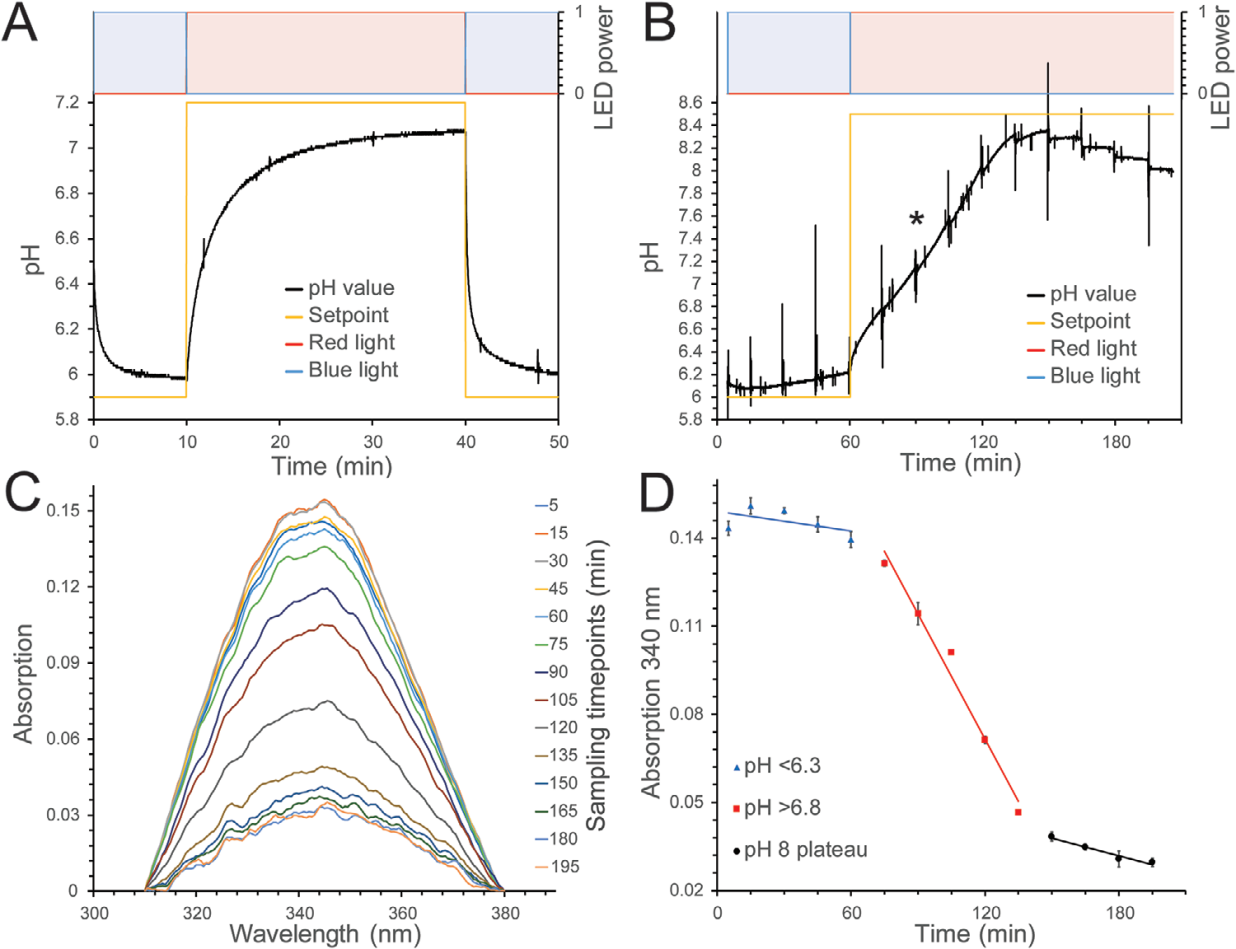
Light color‐controlled pH‐dependent enzymatic reaction of glutamate dehydrogenase. A) pH‐range test of used proteoliposomes. B) pH development during the enzymatic reaction: After changing the light from blue to red at 60 min, the pH increases. The asterisk indicates a turning point at ≈90 min. C) NADH absorption spectra obtained from reaction samples serve as indicators of the reaction progress and reveal a decrease in the absorption maximum over time. D) NADH absorption (panel C) aligned with pH development over time (panel B). Each sampling point is measured in triplicate and shown with SD. Data from one representative experiment of more than three independent experiments is shown.

This enzymatic reaction is pH sensitive and the consumption of NADH is tracked via absorption changes at 340 nm. Thus, the progress of the reaction can be monitored by recording the absorption spectra of the solution at regular time points. The starting pH must be kept close to 6 to hold the reaction progress at a minimal level (see Figure S6, Supporting Information for pH‐dependence of GDH function). After rising to neutral pH, the reaction can run at proper velocity. To enable such pH change in a slightly buffering substrate solution, proteoliposome preparations with the ability to build up a suitable proton gradient are necessary. Suitable preparations were identified by setting the pH setpoint in experiments slightly below 6 and then slightly above 7, and back to below 6, thus testing the pumping power of BPR and BR (**Figure** **3**A). Preparations being able to cover at least one pH unit difference using blue and red light in a range ≈pH 6–7 were further considered.

In a typical experiment, containing all substrates and GDH in the proteoliposome sample, the pH setpoint is set to 6. Figure 3B shows that blue light goes to maximum LED power and BPR pumps protons to keep the pH low. The slow GDH reaction rate at the unfavorably low pH consumes minorly protons and thus raises pH only slightly. After one hour, the pH setpoint is set to 8.5 and red light goes to maximum, powering the proton pumping of BR to move to neutral pH. In this pH regime, the GDH reaction comes to its favorable condition and drives the pH further up, i.e., beyond the pH‐range limit of the proteoliposome system, which is usually visible as a turning point in the pH‐development (see asterisk at ≈90 min in Figure 3B). The pH then reaches a maximum and slowly comes down as the substrates get consumed and the products are accumulating. For monitoring the reaction progress independent of pH, the decrease of NADH absorption at 340 nm was measured in samples taken from the reaction mixture at specific time points. Figure 3C shows spectra in the region of NADH absorption ≈340 nm from reaction samples taken at different time points (see Figure S7, Supporting Information for the extraction procedure). The decrease in NADH absorption at 340 nm initially exhibits small steps, increases in magnitude in the middle section, and subsequently decreases in size toward the end. This is also evident in Figure 3D, which shows the absorption at 340 nm aligned with the pH‐development from Figure 3B. The short spikes every ten minutes in Figure 3B are caused by sample retraction from the vessel for measuring the spectrum in the nanodrop photometer and correspond to a measuring point in Figure 3C. Altogether, this shows the successful control of the enzymatic reaction with light color‐controlled pH adjustments.

In general, a challenging factor of the setup is the buffering capacity of substances in reaction mixtures. Buffering substances, which might be present in the form of reaction substrates and products, must be minimized and optimized to achieve important pH‐changing effects by the proteoliposomes. If there is a proton‐dependent enzymatic reaction involved, like in the example demonstrated in this study, attention must be paid to possible pH influence, e.g., by consumption of protons by the enzyme reaction. On the other hand, such an effect might also be used as an amplifying tool, that increases the reachable pH change (as seen in the GDH reaction, Figure 3B). There is also still potential for increasing the controlled pH range. This can be achieved by optimizing the proteoliposome composition and applied proteoliposome amounts, illumination conditions as well as the use of light‐driven proton pumps with application‐tailored properties, such as fast and efficient pumping, or specific color tuning.

An important feature of the light‐controlled pH system is the good stability of the proteoliposomes and the two proton pumps. It can work for many hours at room temperature and remains functional for weeks when stored at 4 °C. Therefore, it might be useful in low‐volume biotechnological scenarios like chip‐based reaction settings, where enzymatic systems are increasingly applied^[^ ^13^ ^]^ and have to be controlled at the pH level. A similar situation may be found in organ‐on‐a‐chip applications.^[^ ^14^ ^]^ Especially if volumes are so tiny or enclosed that no acid or base can be added, the here presented system is an option for pH adjustment or maintenance against disturbances. Another possible application represents the light‐controlled uptake or excretion of selected molecules across the proteoliposome membrane through proton‐dependent solute transport proteins, which use proton gradients as a driving force.^[^ ^1^, ^15^ ^]^ Furthermore, pH reactive processes and reactions inside proteoliposomes might be controlled. As proteoliposomes are widely used and important systems in various areas of research and applied settings,^[^ ^1^, ^16^ ^]^ having direct control on their inside pH may open new opportunities.

## 3 Conclusion

Modulating or maintaining the local pH in microenvironments without influencing the reaction volume proves valuable, e.g. for studying and controlling proton‐dependent processes. Additionally, the here presented approach can guide pH‐dependent processes, such as enzyme reactions, under specific light conditions. We thus expect that the here presented proteoliposome‐based pH adjustment system controlled by light color represents a powerful tool for various synthetic biology applications and beyond.

## 4 Experimental Section

### Materials

1,2‐dioleoyl‐*sn*‐glycero‐3‐phosphocholine (DOPC) was purchased from Avanti Polar Lipids (Alabaster, AL, USA). Carboxypeptidase Y (C3888‐5MG, 50 U/mg) and glutamate dehydrogenase (10197734001 Roche, beef liver, 15.3 U mg^−1^) were purchased from Sigma. *n*‐octyl‐β‐D‐glycopyranoside (OG) was purchased from Glycon Biochemicals GmbH, Germany.

### Cloning of BPR

The blue‐light absorbing proteorhodopsin Med12 gene (BPR) was synthesized codon‐optimized for expression in *E. coli* (GenScript) based on the published amino acid sequence (GenBank AAY68058.1, PDB: 4JQ6). The BPR gene was cloned using 5′‐HindIII and 3′‐XhoI restriction sites into a version of the pZUDF21 vector and translated a C‐terminal penta‐His‐tag (5H) after a small spacer (LEG – one letter amino acid code), i.e., BPR protein‐LEG‐5H.^[^ ^17^ ^]^ *E. coli* strain C43 (Lucigen) was transformed with the pZUDF21‐BPR construct plasmid.

### Expression of BPR in Escherichia Coli

Luria‐Bertani (LB) medium liquid cultures (100 µg mL^−1^ ampicillin) were inoculated 1:100 with overnight grown pre‐cultures and grown to OD_600_ of 1 at 37 °C. Expression was induced with 0.3 mm isopropyl‐β‐D‐thiogalactopyranoside and 5  µm all‐*trans* retinal (from 100 mm stock solution in ethanol), followed by 4.5 h of further incubation.

### Purification of BPR

BPR‐expressing bacterial cell cultures were centrifuged (8000 x g, 15 min, 4 °C), resuspended in lysis buffer (50 mm Tris‐HCl pH 8, 50 mm NaCl), and lysed by four passes through a Microfluidizer (Microfluidics) at 1,500 bar. The lysate was centrifuged at 10000 x g for 20 min at 4 °C to remove cell debris and unlysed cells. The supernatant was then centrifuged at 150000 x g for 1.5 h at 4 °C in 6 tubes. Pellets were homogenized in 50 mm Tris‐HCl pH 8, 450 mm NaCl and pelleted again (150000 x g, 1 h, 4 °C). This wash step was repeated. Membranes were resuspended in purification buffer (20 mm HEPES‐NaOH pH 7.5, 300 mm NaCl, 10% (v/v) glycerol), and stored at −70 °C or solubilized directly with 3% (w/v) OG for 2 h at 4 °C with gentle agitation (membranes from 0.5 L cell culture in 7 mL). The solubilized membranes were centrifuged at 100000 x g for 45 min at 4 °C and the supernatant was mixed with 0.5 mL bed volume Ni‐NTA Superflow (Qiagen) in 7 mL wash buffer (purification buffer containing 60 mm imidazole). Proteins were bound to Ni‐NTA for 3 h at room temperature (RT) by continuous passage through a gravity flow column. Bound protein was washed with 20 mL wash buffer containing 1% (w/v) OG. Protein was eluted with 1.5 mL elution buffer (20 mm Tris‐HCl pH 8, 150 mm NaCl, 10% (v/v) glycerol, 400 mm imidazole, 1% (w/v) OG), and red colored fractions were pooled. Protein concentrations were determined spectrophotometrically (Nanodrop) at 280 nm using the calculated extinction coefficient 3.3 mL mg^−1^ cm^−1^ (ProtParam; https://web.expasy.org/protparam/).

### Preparation of Purple Membrane from Halobacterium Salinarum

The BR overexpressing *H. salinarum*  S9 strain was grown from a pre‐culture (inoculation 1:50) in L37 medium (4.3 m NaCl, 80 mm MgSO_4_, 10.2 mm Na_3_ citrate, 27 mm KCl, 1% (w/v) Pepton L37 (Oxoid) at 40 °C, 120 rpm (Infors HT Multitron) to an OD_600_ of about 1 (3‐4 d). To induce high BR expression, the rpm was reduced to 80, and the light in the incubator was turned on. After another 3–4 d cells were pelleted (10000 x g, 20 min, RT), washed once with basal salt (4.3 m NaCl, 80 mm MgSO_4_, 27 mm KCl), and cell pellet weight was determined. Cells were lysed by resuspending the pellet in 25 mL H_2_O per g of cells and stirring overnight at 4 °C with 0.02% (w/v) NaN_3_ and DNase I (Sigma, DN25) (1 mg mL^−1^ cells). Cell debris was removed by centrifugation at 4300 x g for 10 min at 4 °C and two times at 7600 x g for 10 min at 4 °C. Purple membrane was pelleted (55000 x g, 1 h, 4 °C), resuspended in H_2_O and centrifuged again as before. Two more washing steps with H_2_O followed (60000 x g, 1 h, 4 °C) with subsequent resuspending of the purple membrane at 1 mL g^−1^ cells with 0.02% (w/v) NaN_3_. Concentration of a 1:5 dilution was determined by measuring the absorption at 568 nm and using the extinction coefficient 2.35 mL mg^−1^ cm^−1^. Samples were stored at 4 °C or frozen at −20 °C. For a detailed purple membrane preparation protocol, see.^[^ ^18^ ^]^

### Reconstitution of Light‐Driven Proton Pumps into Proteoliposomes

BR was solubilized by adding 2.8% (w/v) OG to the purple membrane at 2.88 mg mL^−1^ BR and incubating for 3–6 h at RT with gentle agitation. DOPC in chloroform (10 mg) were dried in a glass vial under a stream of nitrogen and then placed in vacuum at RT overnight. Liposomes were generated by adding 2 mL of dialysis buffer (20 mm Tris‐HCl pH 7.5) to the dried lipid and shaking on an Eppendorf Thermomixer (700 rpm) for 10 min at RT. Liposomes were destabilized by adding 0.75% (w/v) OG and shaking for a further 10 min. 330 µg of solubilized BR and 660 µg of purified BPR were added to the destabilized vesicles. The liposomes were extruded through a 200 nm pore‐size polycarbonate membrane (Mini‐Extruder, Avanti Polar Lipids) by 19 passes. Detergent was removed by dialysis against 2 L of 20 mm Tris‐HCl pH 7.5 at RT overnight (Visking dialysis tubing, 14 kDa molecular weight cut‐off).

### Measuring Device Hardware

The microcontroller (Arduino Nano) was connected to LEDs and a micro pH‐electrode with a self‐built circuit board (schematics shown in Figure S4, Supporting Information). The setup contains 15 blue (VAOL‐5GSBY4, Mouser) and 15 red LEDs (LTL2P3EX2KS, Mouser) to illuminate the sample from different angles and to increase the total light intensity. A 47 Ω resistor was placed in front of each individual LED to match the recommended forward voltage and current, and all LEDs of a specific color were connected in a parallel configuration. The LED arrays were powered by an external power source (PPL36U‐050, Mouser) and controlled by the microcontroller using MOSFETs (MTP3055VL, Mouser), which were connected via 68 Ω resistors. A 1 kΩ resistor was connected in a parallel configuration to each LED array to pull down the current and prevent the flickering of the LEDs when the MOSFET was in off‐state. The micro pH‐electrode (In Lab Ultra‐Micro‐ISM, Mettler Toledo) was connected to the microcontroller using a connector board (Analog pH meter kit, DFRobot). A 10 nF capacitor between the 5 V and ground pins helps to provide a steady power supply to the pH‐meter. Upon calibration with a dedicated Python script (pH_Calibration.py) and buffers of known pH, the resulting analog voltage signal could be converted to pH.

The proteoliposome suspension was kept in a glass test tube (6 × 35 mm, HuberLab), attached to the cooling vessel via a foam material adaptor. The tube was suspended in water, which was cooled to 21 °C by the surrounding flow cooler (Sonoplus KG3, Bandelin) connected to a cooling bath (Julabo F20/H). The flow cooler was enveloped by a tailored 3D printed part, which holds the 30 LEDs in place and directs the emitted light at the sample in the center of the device. The electronics were assembled from the outside and protected by two additional 3D printed parts, which encase the connecting cables. To prevent sedimentation of the proteoliposomes, the measuring setup was placed on a magnetic stirrer plate and magnets of different sizes were placed in the sample and in the cooled water surrounding the sample tube. All 3D printed parts were produced with a Prusa MK3s+ 3D printer and PLA filament.

### Controller Device Software

To control the microcontroller with Python scripts from the user computer, pyFirmata (https://pypi.org/project/pyFirmata/) was installed. First, a calibration script was used, where different solutions of known pH were measured (pH_Calibration.py). The analog signal recorded by the micro pH‐electrode versus the known pH could then be described with a linear fit. The resulting slope and intercept could be used to correlate further readings to the corresponding pH. Once calibrated, a different Python script could be used to either cycle through a set of predefined pH setpoints (pH_PreDefSetpoints.py) or to specify the desired pH setpoint in real time (RT_Controller.py). The color and intensity of illumination were controlled by a combination of proportional (P) and integral (I) feedback.^[^ ^19^ ^]^ The PI‐gains could be fine‐tuned in the script or in real time to achieve the desired behavior of the system. Real‐time adjustments were made by editing the PIDparams.txt file that was used by the software. The Python scripts plot the measured data in real‐time and write all relevant information into a .txt‐file, such as time, current pH, pH setpoint, color, and intensity of illumination as well as P and I gains. The Python code was deposited at the Zenodo open data repository: https://doi.org/10.5281/zenodo.8407647.

### Measuring Procedure and GDH‐Assay

Dialyzed proteoliposome samples were washed four times by centrifugation (200000 x g, 20 min, 4 °C) and resuspension of the pellet with 800 µL 150 mm NaCl solution adjusted to pH 6 to eliminate the buffering compound. After another centrifugation, pellets were resuspended in 150 µL and the proteoliposome suspension was transferred to the measuring vessel that was placed in the measuring device. For testing the pH adjustment power of the preparation, the behavior with setpoints of pH 5.9 and 7.2 was tested (Figure 3A). For GDH‐assay experiments, substrates were added as 5 µL stock solutions adjusted to pH 6, to final concentrations of 1.25 mm α‐ketoglutarate, 5 mm NH_4_Cl, 2 mm NADH, followed by 5–8 µL GDH (from 20 U mL^−1^ in 50% (v/v) glycerol stock solution). The amount of GDH was adjusted based on a control experiment as shown in Figure S6 (Supporting Information) to compensate for small variations in freshly prepared GDH solutions. After mixing, the sample was put into the measuring device with a setpoint of pH 6. Every 15 min a sample of 2 µL was taken from the vessel with a 10 µL Hamilton syringe after mixing with a 250 µL Hamilton syringe. The taken sample was diluted sixfold (1.5 µL + 7.5 µL) with 2% (w/v) OG in water. Three times 2 µL of the dilution were used to measure a UV/Vis spectrum with the Nanodrop spectrophotometer. To compensate for dilution variations, the spectra were normalized to the absorbance at 260 nm (A260), and for better visibility, the NADH peak at 340 nm was extracted by subtracting a linear background factor from 210 to 380 nm (Figure S7, Supporting Information). The peak values were plotted aligned with the pH development. To start the reaction, the pH setpoint was set to 8.5.

### pH‐Assay with BR or BPR Proteoliposomes

Experiments were performed as described previously^[^ ^20^ ^]^ and similar to the method described above with the following differences: 800 µL of proteoliposomes containing 435 µg protein and 10 mg DOPC were probed with the InLab Micro Pro pH electrode (Mettler Toledo) connected to a pH‐meter (SevenCompact, Mettler Toledo). The light source was a white light LED (JANSJÖ, IKEA). Data was collected by the connected software from Mettler Toledo (LabX direct pH 2.3) in Excel. Background drift of pH was subtracted, and peaks were displayed as delta pH.^[^ ^20a^ ^]^

### Nanoparticle Tracking Analysis

Proteoliposome particle size distribution and concentration were measured with a NanoSight NS300 instrument (Malvern Panalytical) equipped with a low‐volume flow‐cell top plate on a 405 nm laser module. Proteoliposomes were diluted 1:1000 in 150 mm NaCl (filtered with a 0.2 µm filter). Five static measurement videos of 60 s were recorded with a sCMOS camera at 25 frames per second and data were analyzed with the nanoparticle tracking analysis software 3.4.

### Carboxypeptidase Digestion Assay

Proteoliposomes or purple membrane (with BR) containing 26.4 µg protein (in the case of BR‐BPR‐proteoliposomes relative to BPR) were adjusted to 20 µL with 150 mm NaCl solution. Then, 20 µL of 100 mm MES‐NaOH pH 6.75 without or with 0.1 mg carboxypeptidase Y were added, and the mixture was incubated overnight at 25 °C. Samples of 1.5 µg protein were mixed with 3 µL of 5x SDS loading buffer (25% (v/v) glycerol, 5% (w/v) SDS, 0.32% (w/v) bromophenol blue, 100 mm Tris‐HCl pH 6.8) and incubated for 5 min at 42 °C prior to loading on a 12% NuPAGE gel (Invitrogen/Thermo Fisher Scientific) and running using MOPS/SDS as running buffer.

## Conflict of Interest

The authors declare no conflict of interest.

## Supporting information

Supporting Information

## Data Availability

The data that support the findings of this study are openly available in [Zenodo] at [https://doi.org/10.5281/zenodo.8407647], reference number [8407647].
