# Supplementary material for: Light Color‐Controlled pH‐Adjustment of Aqueous Solutions Using Engineered Proteoliposomes
Source: Adv Sci (Weinh). 2024 Feb 11;11(15):2307524. doi: 10.1002/advs.202307524 (PMC11022694; doi:10.1002/advs.202307524)
Supplement: Supplementary file 1 — Supporting Information [file ADVS-11-2307524-s001.pdf]

ADVANCED SCIENCE
Open Access

# Supporting Information

for *Adv. Sci.*, DOI 10.1002/advs.202307524

Light Color-Controlled pH-Adjustment of Aqueous Solutions Using Engineered Proteoliposomes

*Daniel Harder, Noah Ritzmann, Zöhre Ucurum, Daniel J. Müller* and Dimitrios Fotiadis**

# Supporting Information

**Light Color-Controlled pH-Adjustment of Aqueous Solutions Using Engineered Proteoliposomes**

*Daniel Harder, Noah Ritzmann, Zöhre Ucurum, Daniel J. Müller,* and Dimitrios Fotiadis**

Daniel Harder, Zöhre Ucurum, Dimitrios Fotiadis
Institute of Biochemistry and Molecular Medicine, University of Bern, 3012 Bern, Switzerland
E-mail: dimitrios.fotiadis@unibe.ch

Noah Ritzmann, Daniel J. Müller
Department of Biosystems Science and Engineering, ETH Zürich, 4056 Basel, Switzerland
E-mail: daniel.mueller@bsse.ethz.ch

Daniel Harder, Noah Ritzmann, Daniel J. Müller, Dimitrios Fotiadis
National Centre of Competence in Research (NCCR), Molecular Systems Engineering, Basel, Switzerland

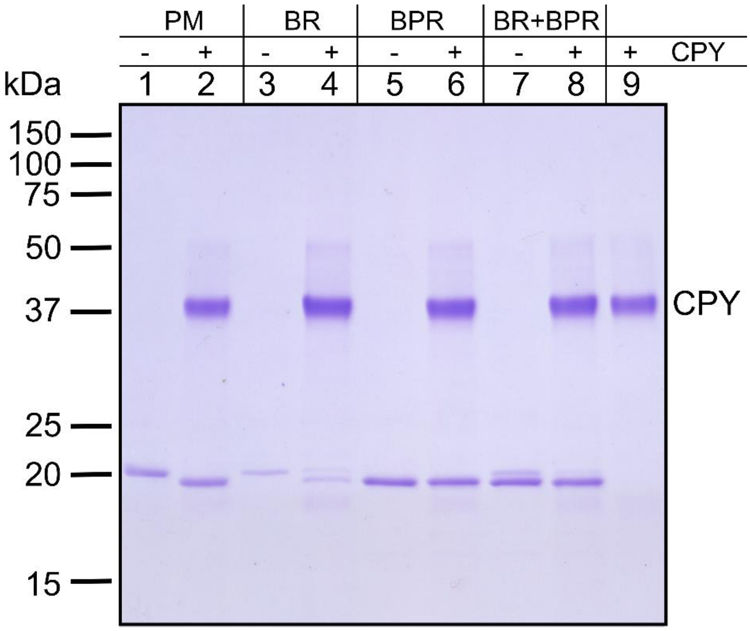


**Figure S1.** SDS-PAGE analysis of purple membrane, and proteoliposomes with bacteriorhodopsin (BR), blue light-absorbing proteorhodopsin Med12 (BPR) or both, i.e., BR and BPR, untreated and treated with carboxypeptidase Y (CPY). Untreated samples: Purple membrane (PM; lane 1), BR- (lane 3) and BPR-proteoliposomes (lane 5), and BR-BPR-proteoliposomes (lane 7). CPY-treated samples: Purple membrane (lane 2), BR- (lane 4) and BPR-proteoliposomes (lane 6), and BR-BPR-proteoliposomes (lane 8). CPY alone was loaded in lane 9. As control, purple membranes were digested, where the extracellular and cytoplasmic sides of BR are accessible to CPY. As seen from lanes 1 and 2, the carboxyl-terminus of BR was completely digested resulting in a band shift towards lower molecular mass. CPY treatment of BR-proteoliposomes also resulted in a clear band shift as evidenced from lanes 3 and 4. BR was mostly accessible to the CPY, i.e., to about two thirds as estimated from the gel band intensities. In contrast, BPR-proteoliposomes were resistant to CPY digestion indicating that the BPR carboxyl-terminus is not exposed to the extravesicular solution. The same was observed when treating BR-BPR-proteoliposomes with CPY. Note that the digested BR migrates together with BPR and cannot be resolved, even under these optimized SDS-PAGE conditions (lane 8). However, efficient CPY digestion was shown previously in the absence of BPR, see lanes 1-4. In summary, the results from the CPY digestion assay indicate that the majority of BR and all BPR were reconstituted into the vesicular membrane as shown in Figure 1A and the observed proton pumping actions of the two light-driven proton pumps are in line with the obtained functional data (Figure S2; Figures 2 and 3A). A 12% NuPAGE gel (Invitrogen/Thermo Fisher Scientific) was used and run using MOPS/SDS running buffer.

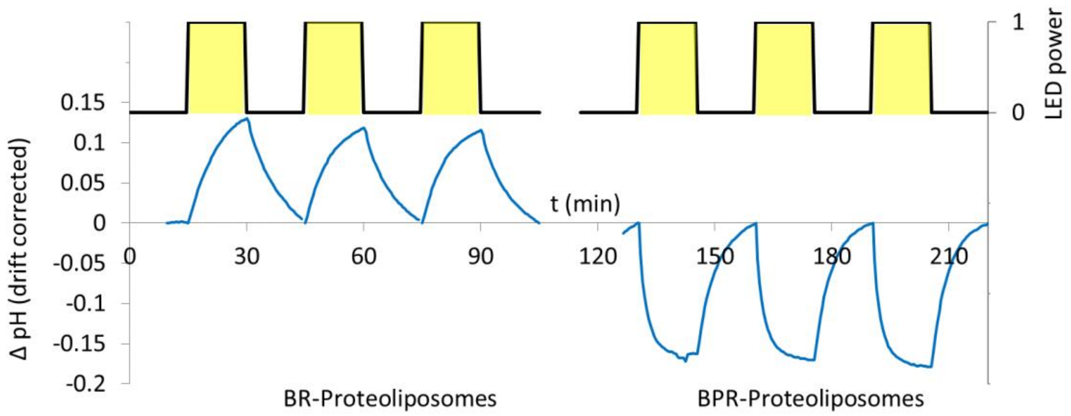


**Figure S2.** pH assay of proteoliposomes with reconstituted bacteriorhodopsin (BR) or blue light-absorbing proteorhodopsin Med12 (BPR). Proton pumping is induced by 15 min of white light followed by 15 min of darkness. The pH peaks of the solution containing BR or BPR proteoliposomes go in opposite directions, which means that the light-driven proton pumps are inserted in opposite directions into proteoliposomes. BR pumps protons to the inside of the proteoliposome, raising the pH of the outside medium, which means that BR is predominantly oriented in the proteoliposome membrane opposite to the physiological situation. BPR pumps protons to the outside of the proteoliposome, acidifying the outside solution, which means that BPR is mainly oriented in the proteoliposome membrane as in the native cell membrane.

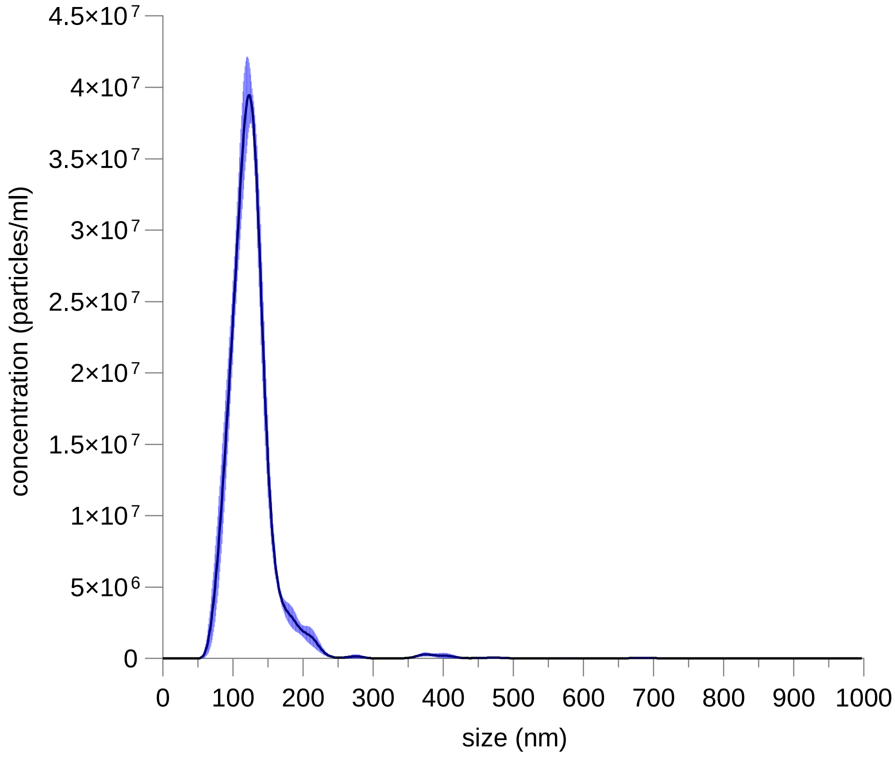


**Figure S3.** Characterization of the bifunctional bacteriorhodopsin (BR)-blue light-absorbing proteorhodopsin Med12 (BPR) proteoliposomes by nanoparticle tracking analysis. Shown is the mean ± standard error (SEM; blue area) of five 60 s movies/experiments. The size distribution shows high homogeneity with a most common particle size (mode) of 125.2 ± 3.1 nm (mean ± SEM) and an average size of 127.8 ± 1.8 nm (mean ± SEM). The particle concentration is 1.2 ± 0.1 $x10^{13}$ particles per mL (mean ± SEM) corresponding to 19.8 ± 1.5 nM (mean ± SEM) in the photoactivity assay (Figures 2 and 3). Displayed data are from five movies/experiments.

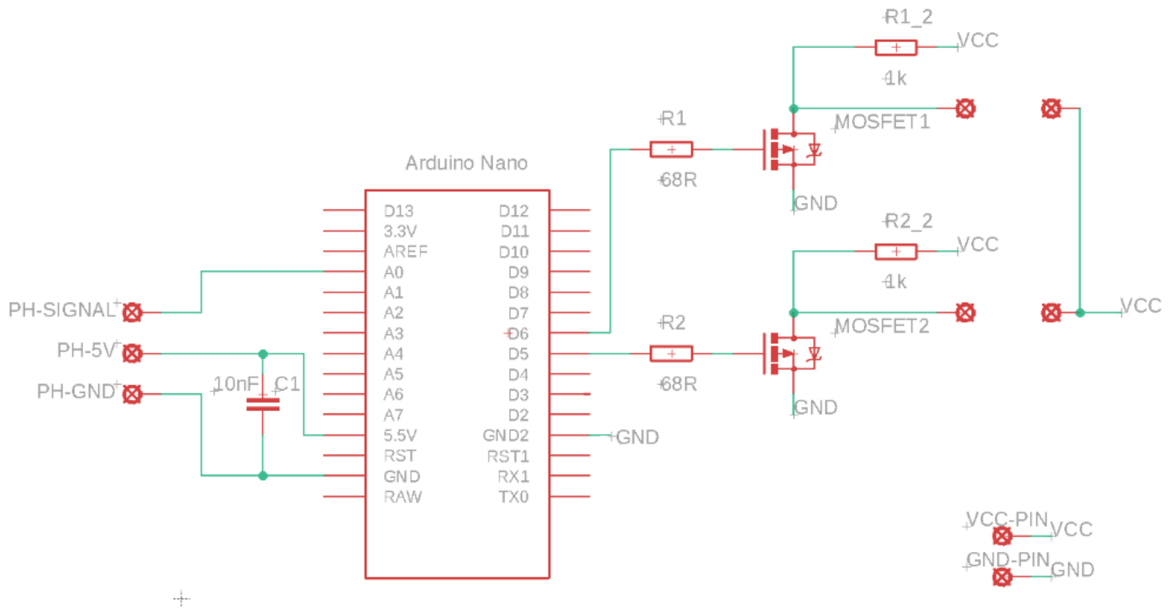


**Figure S4.** Sketch of the electronic circuit of the controller device for feedback loop integration. The Arduino microcontroller is connected via USB to a normal computer with running Python environment employing the code for pH signal interpretation, feedback regulation and control of the LEDs. The python code was deposited at the Zenodo open data repository: DOI: 10.5281/zenodo.8407647

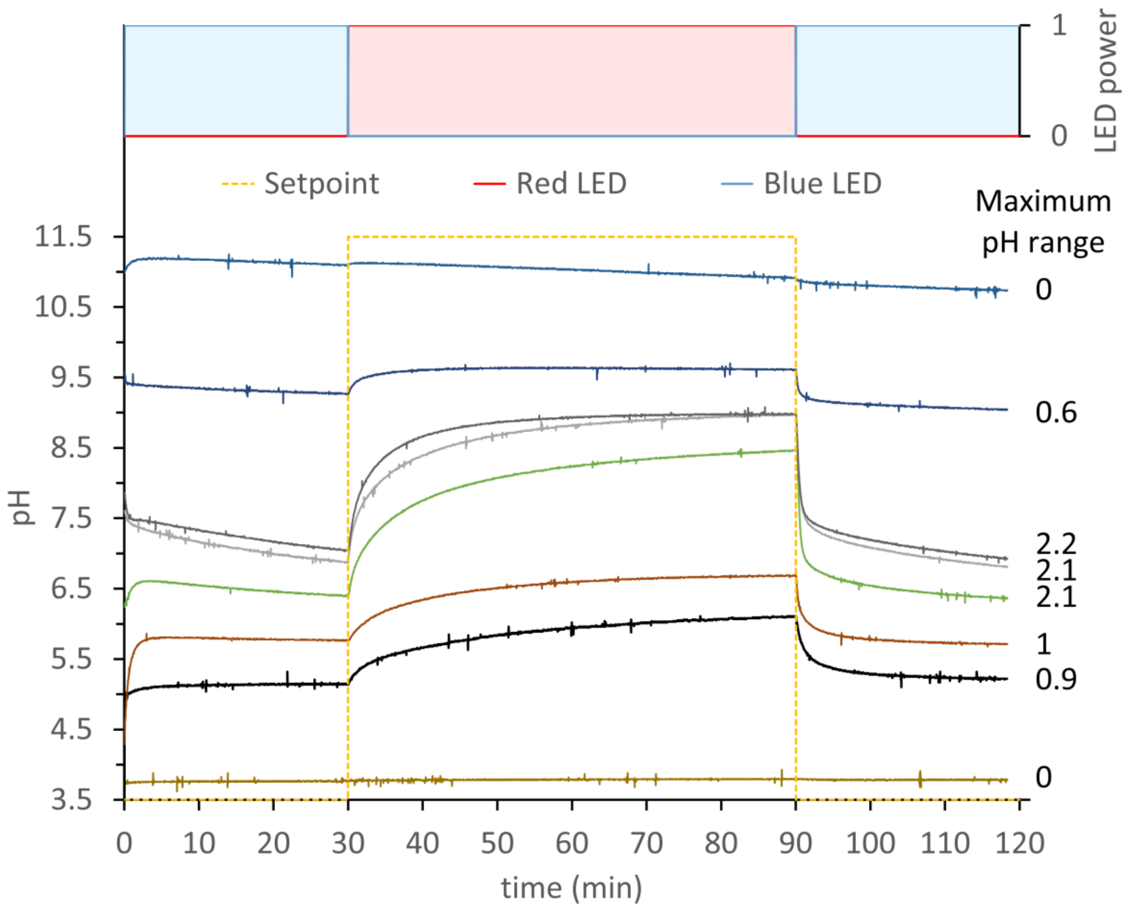


**Figure S5.** Maximal pH ranges and limits of the bifunctional proteoliposome system across the pH spectrum. pH-range test experiments were performed using proteoliposomes at different starting pHs in unbuffered 150 mM NaCl solution, see experiment traces in different colors. First, 30 min blue light was applied to establish the lower pH limit. Then red light was applied for 1 h to bring the pH to the upper limit, followed by 30 min blue light to verify the lower limit. Maximal ranges were achieved when starting around neutral pH, while pH ranges decrease towards acidic and basic starting pHs, even approaching zero (see indicated numbers on the right of Figure).

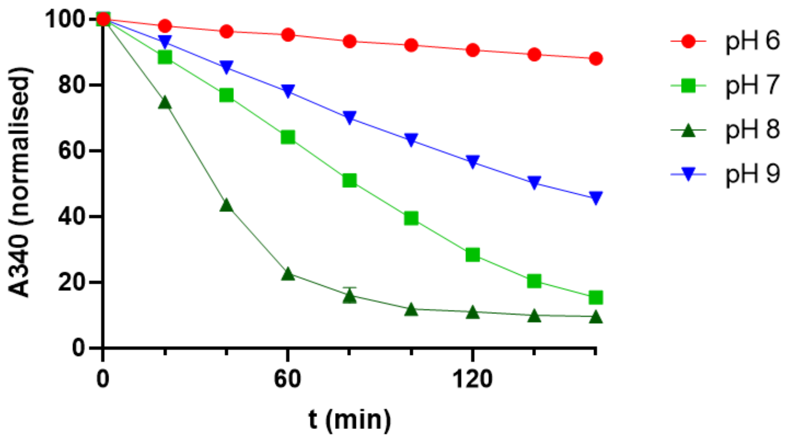


**Figure S6.** pH-dependence experiments of the glutamate dehydrogenase (GDH) reaction. Absorption at 340 nm (A340) is proportional to the substrate NADH concentration and thus reduction of A340 describes reaction progress (i.e., oxidation of NADH to $NAD^+$). The optimum is at pH 8 followed by pH 7 and 9. At pH 6 there is only very low GDH activity. Conditions were similar to GDH-assay experiments (Figure 3; Experimental Section), but in the absence of proteoliposomes and using a buffer mixture at the indicated pH (i.e., MES, HEPES and Tris, each at 100 mM). Data points from the displayed representative experiments at different pHs are means from double determinations ± SD (smaller than symbols for most points).

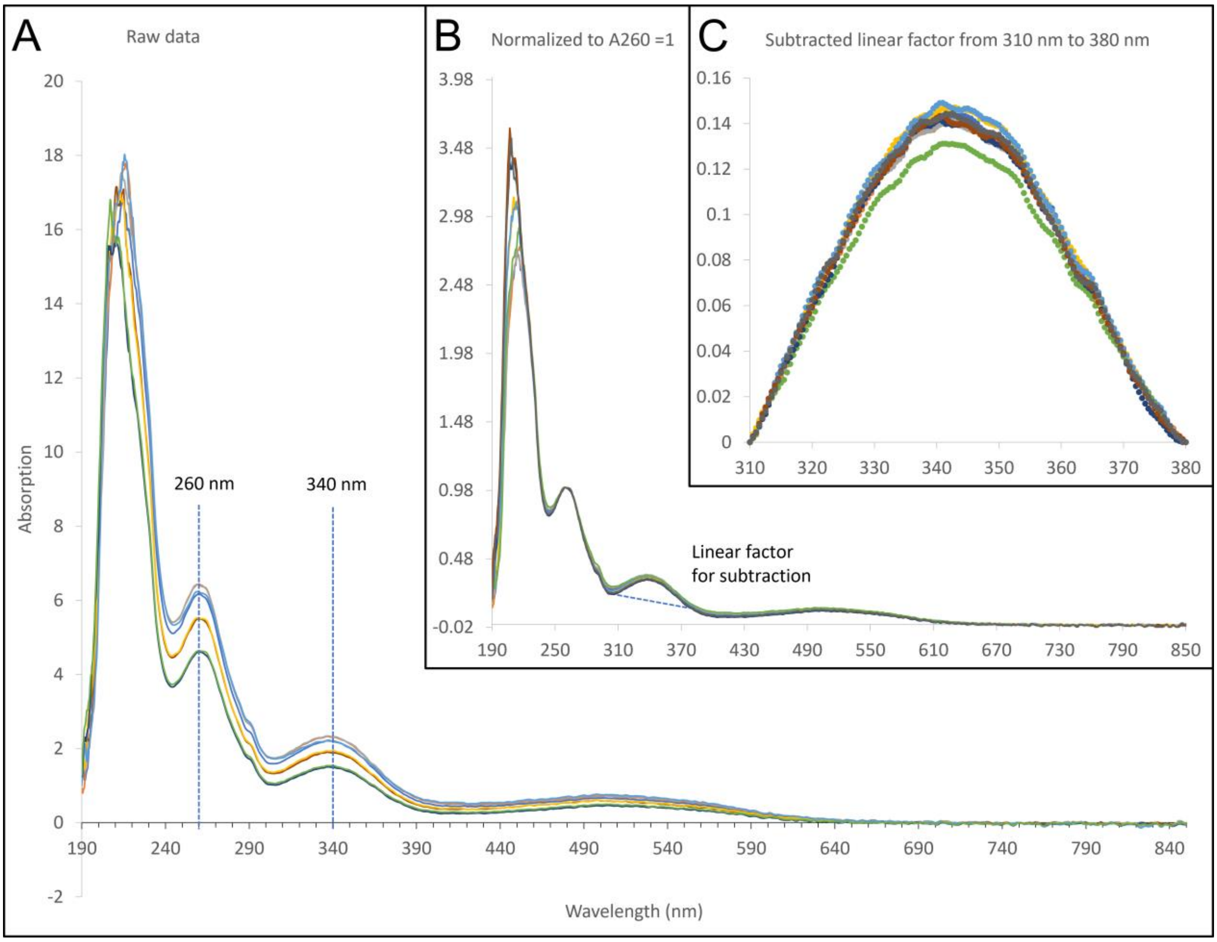


**Figure S7.** Absorption spectrum analysis by normalization and extraction of the 340 nm absorption (A340) peak of glutamate dehydrogenase (GDH)-assay reaction samples. A) Representative raw data from nine measurements of three timepoints shows variance at 340 nm and 260 nm. Differences at 260 nm are due to dilution errors after sample retraction at a timepoint. B) Therefore, absorption spectra were normalized to the absorption at 260 nm (A260) peak, which was set to 1. For better visualization, the A340 peak was isolated from the spectrum by subtracting a linear factor between the upper and lower wavelength limits of the peak from A310 to A380. C) The isolated peak shows much smaller differences in peak height than in raw data.

**Table S1.** Temporal resolution analysis of selected maximum pH ranges. pH changing velocities differ according to the starting pH condition and time subinterval. Velocity is therefore reported in four sectors (see quarters of the maximum range) as it is fast at the beginning and slow at the end of the maximum range. Displayed data are taken from selected

experiments in Figure S5 and the indicated pH range mean values refer to the mean of the upper and lower limit pH values of the corresponding experiments.

| pH range mean | Maxi-mum pH range* | pH changing velocity (delta milli pH/s) | | | | | | | |
|---|---|---|---|---|---|---|---|---|---|
| | | Positive changes (BR) | | | | Negative changes (BPR) | | | |
| | | Quarter of the maximum range | | | | Quarter of the maximum range | | | |
| | | 1 | 2 | 3 | 4 | 1 | 2 | 3 | 4 |
| 9.4 | 0.6 | 4.9 | 2.2 | 0.8 | 0.0 | 17.8 | 10.2 | 1.1 | 0.1 |
| 7.9 | 2.2 | 11.4 | 3.3 | 1.1 | 0.2 | 33.9 | 28.6 | 4.8 | 0.3 |
| 7.4 | 2.1 | 6.8 | 2.3 | 0.9 | 0.2 | 58.9 | 44.2 | 9.8 | 0.3 |
| 5.6 | 1.0 | 2.1 | 0.6 | 0.3 | 0.1 | 10.2 | 5.6 | 2.0 | 0.1 |

* Maximum pH range numbers as indicated in Figure S5 (right side of Figure).
